# High-Frequency rTMS can Improve Depressive Symptoms by Promoting Mitochondrial Fusion

**DOI:** 10.62641/aep.v53i6.1934

**Published:** 2025-12-17

**Authors:** Jingmei Song, Yaru Wang, Simeng Li, Jingyu Yuan, Zhenhui Zhang, Yifeng Pan, Jing Lu, Yuyan Zhang

**Affiliations:** ^1^School of Basic Medicine, Zhejiang Chinese Medical University, 310053 Hangzhou, Zhejiang, China; ^2^School of Life Science, Zhejiang Chinese Medical University, 310053 Hangzhou, Zhejiang, China; ^3^School of Second Clinical Medical College, Zhejiang Chinese Medical University, 310053 Hangzhou, Zhejiang, China; ^4^Vascular Surgery, The Second Affiliated Hospital, Zhejiang University School of Medicine, 310000 Hangzhou, Zhejiang, China; ^5^Department of Psychiatry, The First Affiliated Hospital, Zhejiang University School of Medicine, 310000 Hangzhou, Zhejiang, China

**Keywords:** repetitive transcranial magnetic stimulation, chronic unpredictable mild stress, chronic restraint stress, mitochondrial quality control, mitophagy

## Abstract

**Background:**

Depression is a common and highly prevalent disabling mental disorder. Recent clinical data have shown that repetitive transcranial magnetic stimulation (rTMS) effectively improves depressive symptoms. Mitochondrial quality control (MQC) plays a central role in various psychiatric disorders. However, the relationship between the therapeutic mechanisms underlying rTMS and MQC remains unclear. This study aimed to evaluate the therapeutic effect of rTMS on depression and to investigate the relationship between rTMS and MQC.

**Methods:**

A depression model was established using chronic unpredictable mild stress (CUMS). The rTMS treatment protocol was administered daily for 4 weeks at a frequency of 10 Hz (17 trains of 4 s each, with 15 s intervals), totaling 1000 pulses per day. Each session involved 10 s of stimulation followed by 50 s of rest and was divided into four groups: control, CUMS, CUMS + 10 Hz rTMS, and fluoxetine (FlX)-treated groups (six mice in each group). In this study, we used the open field test (OFT), tail suspension test (TST), sucrose preference test (SPT), and forced swimming test (FST) to assess depression in mice; immunohistochemical staining to observe changes in the prefrontal cortex (PFC), hippocampal neurons, and glial cells; and transmission electron microscopy to detect changes in mitochondrial morphology in the hippocampus.

**Results:**

Our findings suggest that mitochondrial pre-autophagy increased after treatment (LC3Ⅰ/II, F = 34.31,* p *< 0.0001; FIS1, F = 6.666, *p *= 0.0272), hippocampal mitochondrial fusion was enhanced after treatment (NeuN, *p *< 0.0001; c-Fos, *p* < 0.001; MFN1, *p *= 0.0006), and that treatment significantly improved the depression-like behavior of mice in the SPT (*p *= 0.0024) and FST (*p *= 0.0025).

**Conclusion:**

The present study demonstrates that rTMS improves depression-like behavior in mice by promoting mitochondrial fusion and enhancing autophagy.

## Introduction

Depression is a highly recurrent mental disease with an unclear mechanism. Its clinical manifestations include sadness, lack of pleasure, low self-esteem, loss of consciousness, sleep interruption, and loss of appetite [1]. Depression also creates a huge economic burden and is an important cause of global disability [2]. Approximately 30% of patients treated for major depressive disorder (MDD) are treatment-resistant, and the probability of relieving depressive symptoms decreases with medication use after receiving two or more first-line antidepressants. As a safe and well-tolerated noninvasive treatment mode, repetitive transcranial magnetic stimulation (rTMS) improves cerebral cortical excitability through magnetic pulses and weak currents, which have therapeutic effects on depression [3]. However, the mechanism underlying the effect of this induction has not yet been clarified, and research has mainly focused on the synaptic plasticity of nerve cells.

The brain is the most energy-intensive organ in the human body [4]. Brain neurons have a unique polarization and high energy requirement, which relies on specialized mechanisms to maintain energy homeostasis throughout the cell, particularly in the distal axons [5]. Mitochondria are densely distributed in the cells of the central nervous system and can provide most of the energy, affect synaptic plasticity, and are important organelles. Dysfunction of mitochondrial function can impair cognitive function, leading to cognitive impairment and even depression [6, 7].

Mitochondrial quality control (MQC) is an important mechanism for the normal operation of mitochondria. It can regulate the morphology, quantity, and quality of mitochondria through mitochondrial biogenesis, division and fusion, and autophagy to ensure the normal progression of various physiological functions in mitochondria [8]. Studies have examined mitochondrial gene expression in two brain regions associated with depression, namely the prefrontal cortex (PFC), and found that mitochondria-related gene pathways are most pronounced in the PFC [9]. We conducted a series of experiments to explore the relationship between rTMS and MQC systems in the PFC.

## Materials and Methods

### Experiment Animals

Animal care and use were approved by the Animal Care and Use Committee of Zhejiang University.

C57BL6 mouse (8 weeks old) was provided by Zhejiang University Laboratory Animal Center. The mice were fed in standard cages with temperature control (23 ± 1 °C) and humidity control (40%), and were fed in a 12 h standard light-dark cycle. The mice were provided with free access to food and water. They were acclimated for one week before modeling.

### Experimental Grouping

**Fig. 1.**
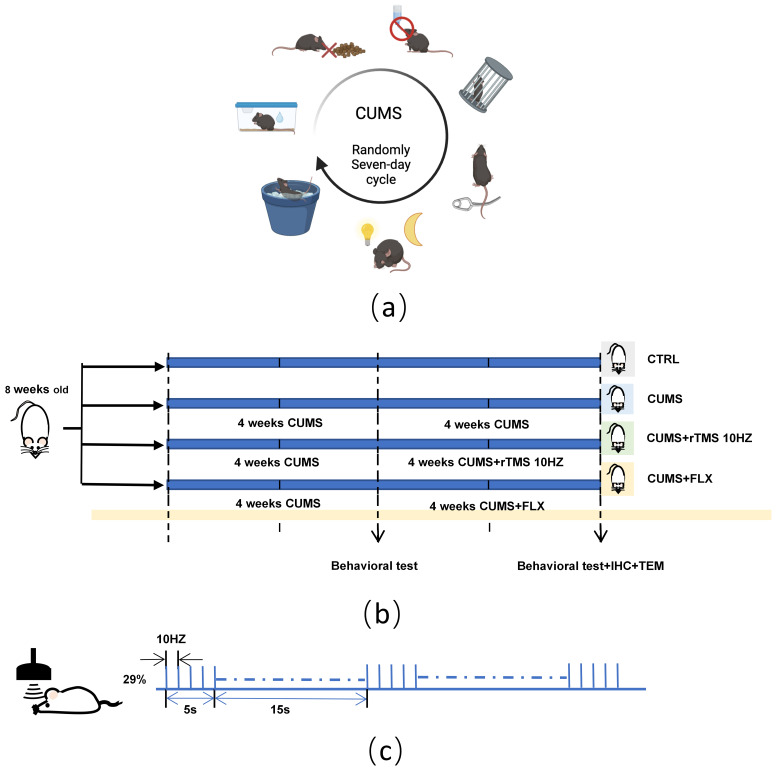
**Schematic of experimental and modelling processes**. (a) Schematic diagram of the CUMS model. (b) Schematic diagram representing the schedule of the experiment. (c) Schematic diagram of the treatment with rTMS. CUMS, chronic unpredictable mild stress; rTMS, repetitive transcranial magnetic stimulation.

The mice were randomly divided into four experimental groups: control, chronic unpredictable mild stress (CUMS), CUMS+10HZ rTMS, and fluoxetine (FlX, Patheon, France, batch number J20050122) treated (six mice in each group). The flow of the experimental treatments for each group is shown in Fig. 1b. FLX is widely used in clinical practice to treat depression. FLX was dissolved in 0.9% saline solution at a concentration of 2 mg/mL. FLX was administered via oral gavage at a dose of 18 mg/kg/day for 28 consecutive days. The control group did not receive any treatment [10].

### Inducing the CUMS Model

A series of different mild stress stimuli were administered in a random order for four weeks: water deprivation for 24 h, fasting for 24 h, 4 h of Cage Tilt (45 °C), tail clamping for 5 min, lights on during the dark phase, swimming in 4 °C ice water for 5 min, Soaked Cage for 4 h (Fig. 1a) [11]. All stimuli were arranged randomly within one week. The stress sequence of each week could not be repeated. The modeling lasted for 4 weeks.

### rTMS Treatment

rTMS was delivered using a commercial stimulator (Magstim Rapid 2; Magstim Company Ltd., Whitland, UK; YRD-CCI, Wuhan, China). During stimulation, mice in the rTMS-treated group were gently restrained manually with the assistance of a plastic cylinder. A circular coil (6 cm in diameter, generating 3.5 T peak magnetic fields) was placed perpendicular to the cortical surface over the projection area of the PFC in mice. All procedures followed the established animal experimental protocols described in previous studies. The stimulation parameters were set as follows: a frequency of 10 Hz (comprising 17 trains of 10 Hz, each train lasting 4 s, with a 15 s interval between trains, Fig. 1c), a pattern of 10 s stimulation followed by 50 s rest (repeated 10 times), and an intensity of 23% of the maximum stimulator output. The treatments were administered daily at the same time [12]. Over a 4-week period, the mice in the rTMS groups received 1000 pulses per day.

### Sucrose Preference Test (SPT)

Before and after the 4-week and 8-week CUMS periods, all mice underwent the SPT to evaluate depression-like behaviors. The SPT procedure included four stages: adaptation, baseline measurement, water deprivation, and final preference test [13]. During the adaptation phase, each mouse was housed individually with two water bottles for one day. In the baseline stage, one bottle contained plain water and the other contained 2% sucrose solution; bottle positions were switched after 12 h. After 24 h of free access to both bottles, fluid consumption was measured by weighing. Sucrose preference was determined as the percentage of sucrose solution intake relative to the total fluid intake (sucrose intake × 100 / total intake).

### Forced Swimming Test (FST)

At the 4- and 8-week time points before and after CUMS exposure, all mice underwent the FST. The test was conducted in a transparent glass cylinder (19 cm in diameter, 27 cm in height) containing water maintained at 24 ± 1 °C, with a water level of 15 cm. This water depth was selected to ensure that the mice could not touch the bottom of the container with their tail or hind limbs. Each animal was gently placed in water for a 6-min session under dim lighting conditions. After the test, the mice were dried and returned to their cages. Immobility was characterized by floating behavior, during which the mice made only the minimal movements necessary to maintain balance and keep their heads above the water. The total duration of immobility during the final 5 min of the test was recorded by two independent observers [14].

### Tail Suspension Test (TST)

At the 4- and 8-week time points of the CUMS procedure, all mice underwent the TST to evaluate depression-like behavior. The TST was carried out following the methodology outlined by Cryan JF *et al*. [15], 24 h after the FST. Each mouse was affixed with adhesive tape to a bar and suspended upside down, with its head positioned approximately 20 cm above the ground. Mouse behavior was continuously recorded for 6 min using a high-definition camera. Immobility was defined as the complete cessation of movement and the absence of struggling. The total duration of immobility during the final 5 min of the test was analyzed by two independent observers who were blinded to the treatment assignments of the animals.

### Open Field Test (OFT)

At 4 and 8 weeks after CUMS exposure, all mice underwent an OFT to evaluate depression-like behaviors. The OFT was performed according to the methodology outlined by Kraeuter AK *et al*. [16], 24 h after the TST. In a dimly lit environment, each animal was gently placed at the center of an open field apparatus (45 cm × 45 cm × 45 cm) to monitor locomotor activity and anxiety-related behavior over a 5-min period. The apparatus was carefully cleaned with 75% ethanol between trials and allowed to dry completely before testing the next subject. At the start of each session, the mice were positioned in the center of the arena and allowed to explore freely. Their movement trajectories were captured using an overhead camera and subsequently analyzed using ANY-maze software (Stoelting Co, USA). The documented parameters included the total distance traveled (indicating horizontal activity) and the distance and time spent in the peripheral zone.

### Tissue Preparation

Twenty-four hours after the behavioral tests, mice were deeply anesthetized with 2% sodium pentobarbital (Sigma-Aldrich, UK, 11715, 50 mg/kg) via intraperitoneal injection and perfused transcardially with ice-cold phosphate-buffered saline (PBS). The brains of some mice were removed quickly and immediately stored in liquid nitrogen for Western Blot (WB). The remaining mice were perfused with 4% paraformaldehyde/phosphate buffer (PFA), then their brains were quickly removed, fixed in 4% paraformaldehyde at 4 °C for 24 h, and frozen and protected in 15% and 30% sucrose until fractional dehydration until they sank to the bottom. Then, they were embedded in optimAI cutting temperature compound (OCT). Coronal brain sections (20 µm) were cut on a freezing microtome (CM1950, Leica Biosystems, Wetzlar, Germany) at –20 °C and stored in Cyto-protection buffer (30% Ethylenglycol, 30% Glycerol in PBS buffer) at –20 °C in the dark for immunohistochemistry (IHC).

### Immunohistochemistry (IHC)

Following dissection, the brains were post-fixed overnight at 4 °C using 4% paraformaldehyde (PFA). Subsequently, they were sequentially cryoprotected by immersion in 20% sucrose (prepared in 4% PFA) and then in 30% sucrose (dissolved in 0.1 M PBS). Using a microtome, 30-µm-thick coronal sections were obtained. For antigen retrieval, the sections were subjected to incubation in 0.01 mol/L citrate buffer (pH 6.0) at a high temperature, blocked in 2% (w/v) BSA (Sigma), and then exposed overnight to the following primary antibody mixtures: anti- c-Fos (1:200, Abmart, TU312995s), anti-FIS1 (1:500, Proteintech, 10956-1-AP), anti-MFN1 (1:500, Proteintech, 66776-1-IG), and anti-NeuN (1:1000, Abmart, T55515S) at 4 °C. Following primary antibody incubation, the sections were incubated with biotinylated secondary antibodies (1:200, Affinity, S0002; 1:200, Affinity, S0001; 1:2000, FUDE, FD0128) and counterstained with hematoxylin. The staining procedure was performed using a DAB kit (OriGene Technologies, Inc., Beijing, China) following the manufacturer’s instructions. For quantitative assessment, positively stained cells and the total number of cells in each field were counted to determine the proportion of positive cells. Additionally, the integrated optical density (IOD) and the corresponding area were measured, and the average optical density was derived by dividing the IOD by the area.

### Transmission Electron Microscope (TEM)

Following dissection, the tissue specimens were promptly immersed in a 2.5% glutaraldehyde solution for fixation for 12 h. After fixation, the samples were rinsed with phosphate buffer (pH 7.4) and treated with 1% osmium tetroxide in the same buffer. Dehydration was performed by using a graded series of ethanol solutions. Subsequently, the tissues were rinsed with propylene oxide and embedded in epoxy resin. Using an ultra-microtome equipped with a glass knife, semi-thin sections (approximately 1–2 µm) and ultrathin sections (around 70 nm) were prepared. Ultrathin sections were mounted on copper mesh grids and double-stained with uranyl acetate followed by lead citrate. Finally, the prepared specimens were imaged using the transmission electron microscope (Philips, Netherlands, TECNAI-10) operating at an accelerating voltage of 60 kV.

### Western Blot (WB)

Fresh hippocampal tissue was mechanically disrupted using a homogenizer (Jingxin, Shanghai, China) in radioimmunoprecipitation assay (RIPA) lysis buffer (Beyotime, Shanghai, China). The homogenate was centrifuged at 12,000 ×g for 20 min, and the supernatant was collected. The total protein content in the supernatant was determined using a bicinchoninic acid (BCA) assay kit (Pierce Scientific). Subsequently, proteins from each sample were resolved by electrophoresis and transferred onto polyvinylidene fluoride (PVDF) membranes. The membranes were blocked with 5% non-fat milk for 2 h, followed by an overnight incubation with primary antibodies targeting MFN1 (1:3000, Proteintech, 66776-1-IG), FIS1 (1:2000, Proteintech, 10956-1-AP), C-Fos (1:500, abmart, TU312995s), and β-actin (1:50,000, HUABIO, EM21002). After washing, the membranes were probed with horseradish peroxidase-conjugated secondary antibodies, including goat anti-mouse (1:5000; Affinity, S0002) and goat anti-rabbit (1:5000; Affinity, S0001), for 2 h at room temperature. Protein signals were visualized using an ImageQuant LAS4000 mini system (GE Healthcare, Buckinghamshire, UK), and band densities were analyzed using ImageJ software (version 1.54k, NIH, USA). To account for potential sampling variability, the relative expression levels of target proteins were normalized to β-actin.

### Statistical Analyses

The data are presented as the mean ± Standard Error of the Mean (SEM). The results were analyzed using Student s’ test for comparison between two groups or one-way Analysis of Variance (ANOVA) for multiple-group comparison (GraphPad Prism 9, San Diego, CA, USA) and one-way or two-way ANOVA followed by Tukey’s post-hoc test for multiple comparisons. All statistical analyses were performed using GraphPad Prism software (GraphPad Prism 9, San Diego, CA, USA), and statistical significance was defined as *p* < 0.05.

## Results 

### CUMS Induces Depressive-Like Behavior

**Fig. 2.**
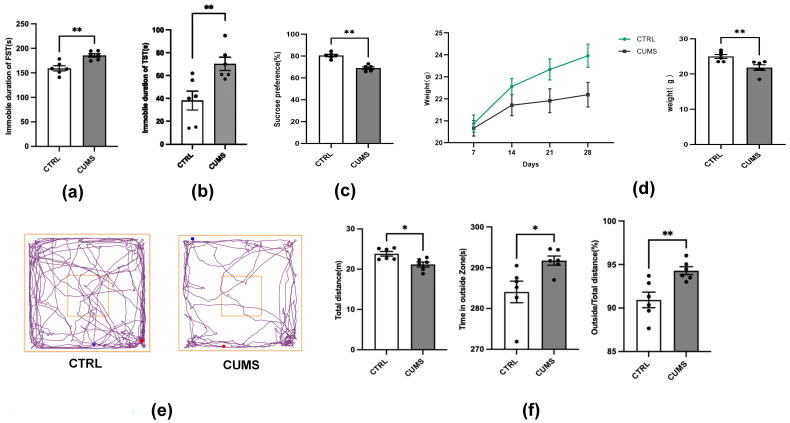
**Four-week CUMS-induced depression-like behaviors in mice**. (a) The results of FST after CUMS. (b) The results of TST after CUMS. (c) Sucrose preference after CUMS. (d) The effects of CUMS on body weight. (e) Trajectories in the OFT after CUMS. (f) Total distance traveled and the time spent on the outside in OFT after CUMS. *p < 0.05; **p < 0.01; ns, not significant. The values represent mean ± SEM. n = 6. CUMS , chronic unpredictable mild stress; FST, forced swimming test; TST, tail suspension test; OFT, open field test.

The first step was to rule out modelling failures. In the fourth week of modelling, by comparing the data of the TST, FST, SPT, and OFT between the control and modelling groups, we found that the mice in the modelling group had a longer resting time in the TST and FST (t = 3.193, *p *= 0.0096; t = 4.192, *p *= 0.0019; Fig. 2a,b), showing despair. In the SPT, the mice showed a decreased preference for sucrose (t = 8.345, *p *= 0.0022; Fig. 2c) and a lack of pleasure. Combined with the curve of change in body weight (Fig. 2d), we found that the body weights of mice in the modelling group were significantly lower than those of mice in the control group. In the OFT, the total traveling distance of the model group significantly decreased, whereas the peripheral traveling path significantly increased (t = 3.075, *p *= 0.0117; t = 2.2260, *p *= 0.0239; t = 3.342, *p *= 0.0075; Fig. 2e,f), indicating a reluctance to remain in the center. The above experiments show that the mice were modelled by CUMS, and the depression-like mice were successfully modelled.

### rTMS Improves Depressive-Like Behaviors

**Fig. 3.**
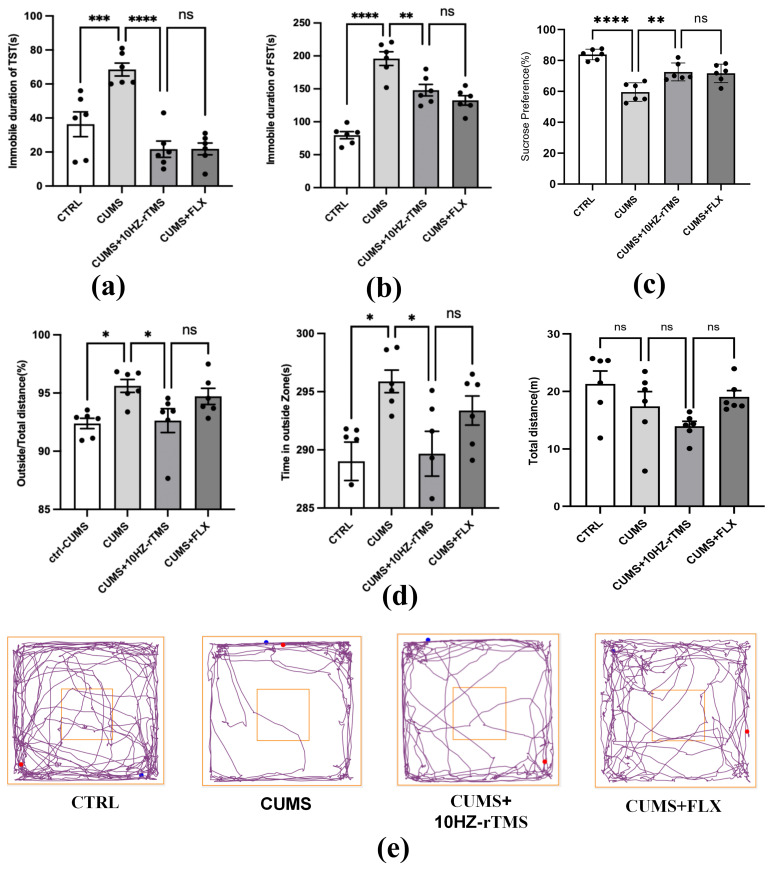
**Effect of rTMS and FLX treatment on TST, FST, SPT, and OFT in CUMS mice**. (a) The immobility time in TST. (b) The immobility time in FST. (c) Sucrose preference. (d) Total distance traveled and the time spent on the outside in OFT. (e) Trajectories in the OFT. *p < 0.05; **p < 0.01; ***p < 0.001; ****p < 0.0001; ns, not significant. The values represent mean ± SEM. n = 6. FLX, fluoxetine; SPT, sucrose preference test.

After rTMS and FLX intervention, depression-like behaviors were significantly reduced. Mice in the TST treatment group showed alleviation of despair-like behaviors with a higher frequency of locomotion (F = 45.60, *p *= 0.0002; Fig. 3). In the FST, mice in the treatment group showed a stronger desire to survive in water (F = 48.00, *p *= 0.0025; Fig. 3b). In the SPT, mice in the treatment group had a stronger preference for sugar water and were more desirous of obtaining pleasure from it (F = 11.28, *p *= 0.0087; Fig. 3c). Meanwhile, the rTMS treatment group demonstrated a greater willingness to explore the center, with shorter distances traveled in the periphery and a higher total distance of movement (F = 4.892, *p *= 0.0366, F = 4.66, *p *= 0.0375; F = 2.858, *p *= 0.5479; Fig. 3d,e). Taken together, these data suggest that rTMS treatment can improve depression-like behaviors in mice.

### rTMS Alleviates Neuronal Damage in the mPFC

**Fig. 4.**
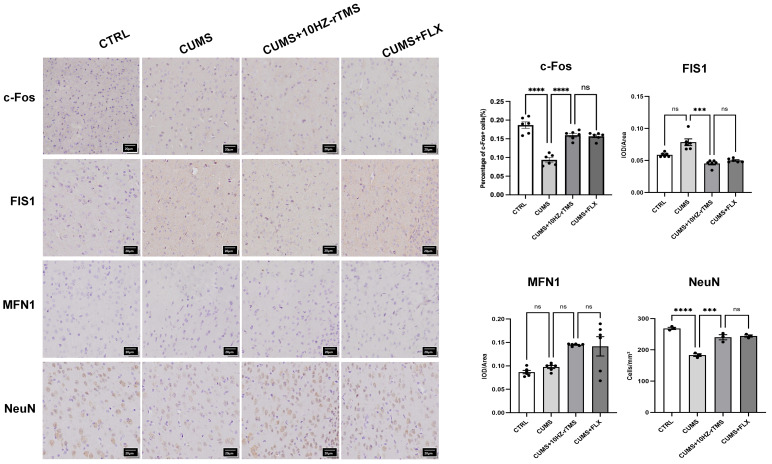
**Effect of rTMS and FLX treatment on c-Fos, FIS1, and MFN1 protein expression and neuronal damage in the PFC of CUMS mice**. ***p < 0.001; ****p < 0.0001; ns, not significant. The values represent mean ± SEM. n = 6. Each cell of the scale represents 20 µm. PFC, prefrontal cortex.

As shown by IHC (Fig. 4), the number of neurons in the medial PFC of mice in the depression model was significantly reduced (F = 5.040, *p *= 0.0253), and the c-Fos protein level was decreased (F = 40.68, *p* < 0.0001). This indicates that some of the neurons were damaged by chronic stress, and the reward and emotion-related signaling capacity was reduced. This led to depression-like behaviors in the mice. After treatment, from the immunohistochemical images, we found that compared with the model group, the number of neuronal cells in the rTMS-treated group increased (F = 47.130, *p* < 0.0001, *p *= 0.0003), the content of c-Fos increased (F = 40.68, *p* < 0.0001), and the content of FIS1 decreased (F = 23.70, *p *= 0.0002); there was no significant difference in the results of MFN1. These two data points in the FLX-treated group showed a trend similar to that of the rTMS-treated group.

### rTMS Activates the MQC and Promotes Mitochondrial Fusion

**Fig. 5.**
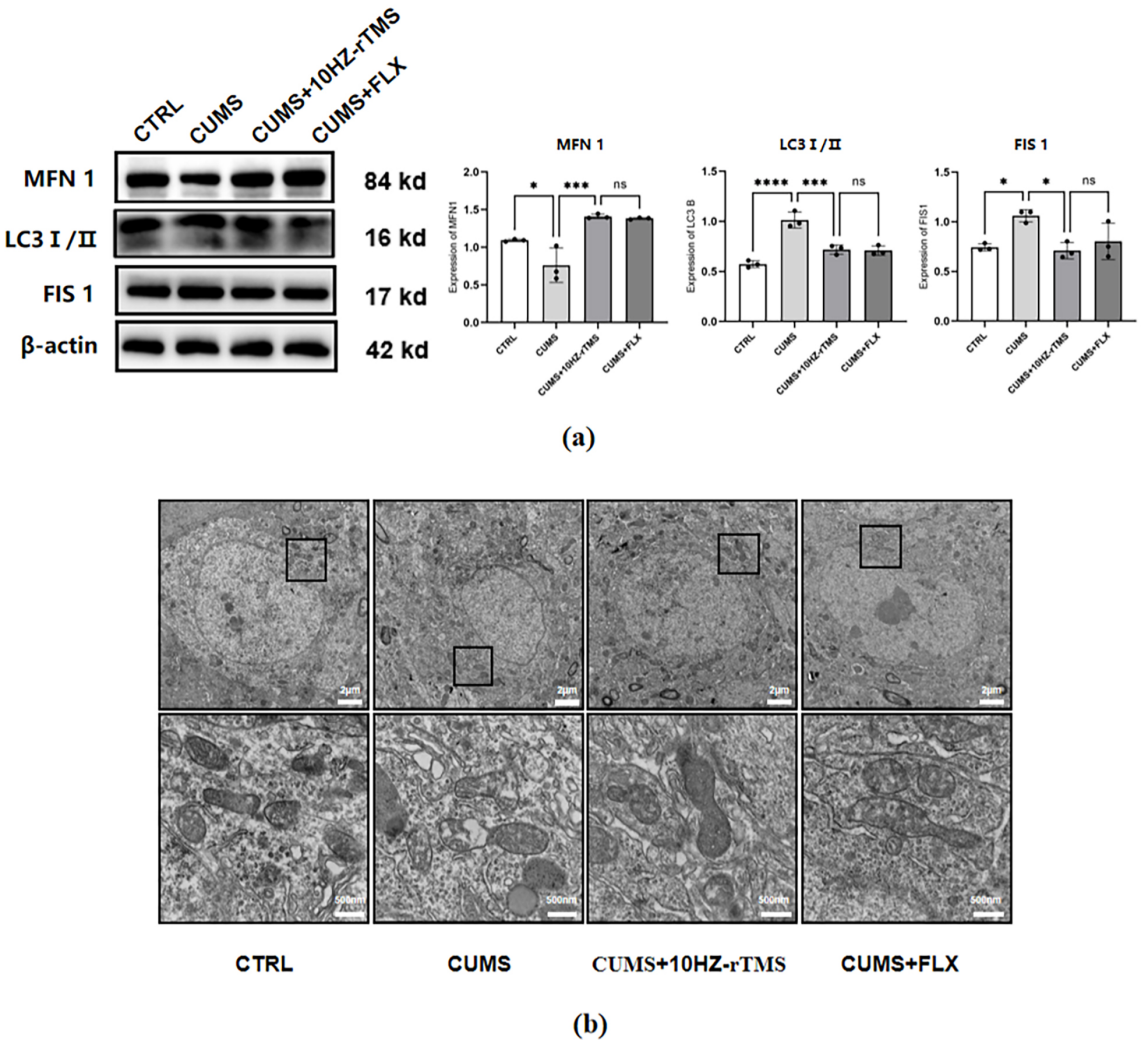
**Effect of rTMS and FLX treatment on MFN1, LC3, FIS1, and protein expression and MQC in CUMS mice**. (a) Expression levels of MQC-related proteins. (b) Mitochondrial morphology in TEM rTMS and FLX treatment ameliorates mitochondrial damage and promotes autophagosome formation in the cerebral cortex of CUMS mice. TEM showed that mitochondria were normal in the CTRL group, and WB showed that FLX may improve depressive symptoms through mitochondrial autophagy. Mitochondria were severely disrupted in CUMS group. Several autophagosomes were observed in CUMS+rTMS 10 Hz and CUMS+FLX groups. *p < 0.05; ***p < 0.001; ****p < 0.0001; ns, not significant. MQC, Mitochondrial quality control; TEM, Transmission Electron Microscope; WB, Western Blot.

We examined the expression of proteins related to the mitochondrial control system (Fig. 5a). For LC3, the protein level of LC3I/II was mainly detected, and there was a difference between the control group and the CUMS modelling group (F = 34.31, *p* < 0.0001), as well as a difference between the CUMS modelling group and the rTMS treatment group (F = 34.31, *p *= 0.0008), and no difference between the rTMS and Flx treatment groups (F = 34.31, *p* > 0.99). In the detection of MFN1, there was a difference in the level of MFN1 between both the modelling group and the control group (F = 20.57, *p *= 0.0325), whereas after rTMS treatment, we found a significant increase in the level of MFN1 (F = 20.57, *p *= 0.0006).

Secondly, FIS1, an indicator of mitochondrial division, was upregulated after modelling (F = 6.666, *p *= 0.0272) and decreased after treatment (F = 6.666, *p *= 0.0160). In addition, under an electron microscope, mitochondria in the CUMS group showed varying degrees of swelling, reduced cristae density, enlarged membrane gaps, and mitochondrial fragmentation (Fig. 5b). Notably, the mitochondria in the rTMS group were clearly encapsulated by autophagic lysosomes and were in the process of fusion. This demonstrates that rTMS treatment may be able to protect cells by initiating MQC and promoting mitochondrial autophagy. The above experiments indicated that the MQC system was activated after rTMS treatment, which may further increase the level of mitochondrial fusion by promoting mitochondrial fusion and inhibiting mitochondrial division, preventing excessive division of mitochondria, and maintaining normal mitochondrial ultrastructure and morphological function.

## Discussion 

The repeated presentation of the same stressors usually leads to adaptation; however, adaptation can be prevented by presenting different stressors in an unpredictable sequence [13]. The CUMS model overcame this adaptation and produced long-lasting depression in the animal groups. This method of chronic unpredictable stimulation consists of a series of tests, such as water or food deprivation, cage tipping, tail pinching, diurnal reversal, swimming in ice water, and cage immersion. The most obvious feature of animal models is anhedonia, an underlying symptom in patients with depression. Initial modelling could produce several depressive symptoms, such as increased immobility time in the TST and FST, reduced sucrose preference in the SPT (sucrose preference was calculated based on sucrose consumption as a proportion of total fluid consumption), and reduced time spent in the periphery in the OFT. This finding is consistent with the conclusions of the present study. Currently, the CUMS model is widely used and researched to study the underlying mechanisms, etiology, and new animal models of depression, making it a reliable and replicable animal model [13, 17]. Therefore, in this study, we established a depression model using CUMS.

Clinical research indicates that high-frequency rTMS is a non-invasive therapeutic method for delivering magnetic pulses that can effectively treat depression [18]. Chronic stress promotes the atrophy of mPFC neurons and a reduction in synaptic number and function, which impairs or prevents neuronal plasticity, leading to a sustained decrease in brain signaling [19]. In depression, the PFC of the brain is consistently damaged [20], and the mechanism of action of rTMS in the mPFC has been a major target of research for therapeutic applications in depression [21]. Clinical research has confirmed that application of high-frequency rTMS to the PFC produces antidepressant effects [22]. Additionally, studies have suggested that rTMS may induce synaptic plasticity and promote neuronal repair by altering gene expression, neurotransmitters, and receptor functions associated with synapses in stimulated areas [23]. Therefore, we used the PFC as the main detection site in this experiment. We will follow this with cortex-related testing to determine the differences in the response to magnetic stimulation in areas of different depths.

Dysfunction of mitochondrial autophagy and abnormal over-division of mitochondria may be related to the development of depression [24], and mitochondrial autophagy is an important process in maintaining mitochondrial homeostasis, which has been shown to have a mitigating effect on depression, possibly by maintaining mitochondrial homeostasis [25, 26]. Previous studies have indicated that CUMS may adversely affect mitophagy caused by mitochondrial fragmentation in hippocampal dentate gyrus neurons [27]. Research has shown that rTMS can exert a powerful neuroprotective effect, potentially improving the local neuronal microenvironment by maintaining mitochondrial integrity in the peri-infarct area [28].

Although rTMS has been extensively explored in both clinical and basic research for the treatment of depression, and it has been validated that rTMS affects the MQC, it remains unclear whether it can influence depression through its effect on the MQC. Therefore, this study employed the OFT, TST, SPT, and FST to assess the depressive state in mice. Immunohistochemical staining was used to observe changes in neurons and glial cells in the PFC and hippocampus. Western Blot analysis was conducted to observe the expression levels of MQC-related proteins. Transmission electron microscopy was employed to detect changes in the mitochondrial morphology of the hippocampus.

Our results showed that rTMS could ameliorate depressive-like behavior in mice by affecting mitochondrial autophagy: the rTMS-treated group had mitochondrial structures that were fused compared to the CUMS-modelled group. Moreover, we observed a significant increase in the number of neurons in the PFC of the treated mouse group compared to the depression-modelled group by the IHC technique. Therefore, rTMS may ameliorate depressive symptoms by affecting the mitochondrial control system and promoting mitochondrial fusion, particularly in the PFC.

In this study, we used four behavioral experiments, the TST, FST, SPT, and OFT, to assess depression-like behavior in mice. rTMS, as a clinically used and effective non-invasive treatment for depression, modulates cortical excitability in the range of a few minutes to hours and has a clinical effect that can be sustained for a few weeks to months [29]. In addition, it can be very effective in improving an organism’s depression [30, 31], an increase in the resting time of mice in the TST and FST, a decrease in preference for sugar and water in SPT, and a decrease in the desire to explore the surroundings in OPT. Our findings show that this result is consistent with the expected results [32, 33]. Thus, the effect of rTMS on depression was confirmed in the present study.

FLX is a selective 5-hydroxytryptamine reuptake inhibitor (SSRI) that inhibits 5-HT reuptake into the synaptic gap [34]. It is often used to treat depression-like mood and pleasure-deficit disorders. Beside it is a first-line antidepressant, which can modulate synaptic plasticity through the ERK1/2-NF-κB pathway, by affecting MMP-9, etc., to alleviate depression-like symptoms [34, 35]. FLX increases the LC3-II/I ratio to promote mitochondrial autophagy, thereby exerting a cytoprotective effect [24]. LC3 is an autophagy-associated protein [36], a second modifier necessary for late autophagosome formation, and LC3-I covalently binds to phosphatidylethanolamine in response to ATG7 and ATG12-ATG5-ATG16L to form LC3-II, which binds to autophagosome membranes [37, 38]; MFN1 is located in the outer membrane of the mitochondrion and is associated with mitochondrial fusion in response to the mitochondrial fusion levels [39]; FIS1 regulates mitochondrial fission indirectly through lysosomal Rab7 GTP hydrolysis [40] and is associated with mitochondrial segregation [41]. In this study, the protein level of neuronal LC3-II in mice treated with rTMS was elevated, which was consistent with the experimental results of the FLX-treated group, demonstrating a therapeutic effect similar to that of the drug-treated group.

MQC mechanisms include mitochondrial autophagy (mitophagy), fission and fusion processes, the mitochondrial unfolded protein response (UPRMT), mitochondrial biogenesis, and intercellular mitochondrial transfer [40]. Under external stress, mitochondria release molecules that promote cell death or generate toxic reactive oxygen species (ROS), the key mediators of cellular death. Thus, timely activation of mitochondrial autophagy via MQC is essential for clearing impaired mitochondria [42]. At the organelle level, mitochondria with mild damage may recover their functional capacity through fusion with their healthy counterparts or segregate damaged components via fission. If fission or fusion fails to restore functionality, autophagy is triggered to remove defective organelles. The degraded mitochondrial elements are replenished by the synthesis of new proteins and lipids. At the molecular level, mitochondria elicit the transcription of specific chaperone proteins and proteases by dispatching UPRMT signals to the nucleus. Intercellular mitochondrial transfer represents a recently identified MQC process that contributes to the restoration of the mitochondrial network at the cellular level. Hence, mitochondrial fission, fusion, and dynamic movement are integral to the mitophagy process [42]. MFN1 is located on the outer mitochondrial membrane and is associated with mitochondrial fusion, responding to the level of mitochondrial fusion [39]. FIS1 indirectly regulates mitochondrial fission through lysosomal Rab7 GTP hydrolysis [40], and is associated with mitochondrial detachment [41]. Mitochondrial fusion is mediated by the coordinated action of three mitochondrial fusion proteins, MFN1, MFN2, and optic nerve atrophy 1 (OPA1) [43]. MFN1 and MFN2 are localized in the outer mitochondrial membrane and fused in a GTPase-dependent manner, followed by OPA1, which is localized in the inner mitochondrial membrane and fused in a GTPase-dependent manner [44]. The process of mitochondrial fission involves mainly the participation of proteins such as Drp1, FIS1, Mff, Mid49, Mid51, etc. [45].

The transcription factor c-Fos, encoded by the Fos gene, is commonly used as a functional indicator of neuronal activation in neuroscience research [46]. Upon stimulation, c-Fos assembles into the heterodimeric complex activator protein-1 (AP-1), which can partner with additional DNA-binding proteins and subsequently attach to promoter regions of various “late-response” or target genes [47]. Baseline c-Fos expression was lower in depressed mice than in normal controls. However, following the administration of FLX and rTMS, a marked elevation in c-Fos expression was observed in these mice. NeuN is a common antigen found in various vertebrates [48]. Almost all neuronal cell types of the central and peripheral nervous system, including small interneurons, are immunoreactive to NeuN. Therefore, NeuN is considered a sensitive and specific neuronal cell marker [49]. Our study showed that NeuN expression was significantly elevated in the rTMS treatment group, consistent with the effects of the FLX treatment group.

In this experiment, mice treated with rTMS showed a significant increase in mitochondrial autophagy compared with the depression modelling group, suggesting an elevated level of mitochondrial autophagy after rTMS treatment, which restored the mitochondrial disorders that occur in depression, consistent with our expected results. Based on our results, we speculated that rTMS may alleviate depression-like behavior by modulating mitochondrial autophagy. However, rTMS, as a physical factor therapy, remains to be resolved as to which signal transduction pathway it is used through for mitochondrial regulation and how to determine the intensity of its use for better clinical application.

## Conclusion

Our study revealed a part of the mechanism of rTMS in the treatment of depression: rTMS affects mitochondrial autophagy, eliminates damaged mitochondria, improves mitochondrial fusion, and reduces cell death, while increasing the activity and number of neurons in the PFC of mice. We hope that this study will provide a new way to treat depression in addition to drugs.

## Availability of Data and Materials

The authors confirm that the data supporting the findings of this study are available within the article.
